# Reproductive power matters: aligning actions with values in global family planning

**DOI:** 10.1080/26410397.2022.2082353

**Published:** 2022-07-29

**Authors:** Christine Galavotti, Sara Gullo

**Affiliations:** a Senior Program Officer, Bill & Melinda Gates Foundation, Seattle, WA, USA.; b Independent Consultant, Atlanta, GA, USA

**Keywords:** family planning, reproductive power, reproductive agency, reproductive justice, sexual and reproductive health and rights, social determinants

## Introduction

The COVID-19 pandemic has exposed and compounded existing inequities, with disparities in full recovery from the disease and death affected by geographic, racial, social, and economic status.^1^ In the area of sexual and reproductive health, and particularly for adolescents in the global South, school closures have reduced access to comprehensive sexuality education, and lockdowns and disruptions in access to family planning (FP) services have led to increases in sexual harassment, gender-based violence, and unwanted pregnancies.^2^ These facts, as well as the occurrence of gross imbalances in access to vaccines, catastrophic losses in income, and increased hunger, have inspired a re-examination of the models of development that have failed so many, with more and more global health actors waking up to what justice activists and scholars have been pointing out for many years – that health cannot be extracted from the economic, social, and political context in which it is produced, or inhibited. Leaders like Loretta J. Ross and Dazon Dixon Diallo from the Reproductive Justice movement in the United States have long called for this realignment,^3^ but the emergence of these concepts in the broader global health discourse is more recent and needs to be nurtured. In addition to the latest report from the High-level Commission on the Nairobi Summit on ICPD+25,^2^ this rethinking has included a call from Donald Berwick, a leading authority on health care quality and improvement, for the health community to collectively turn our focus to the “moral determinants of health” – in other words, to actions that are truly aligned with our values to improve human health and well-being. This call is rooted in the reality that efforts to improve health and well-being have illogically and drastically underinvested in addressing societal and structural factors, despite robust evidence that circumstances outside of health care are largely responsible for health and well-being.^4^

What does this mean for those working in global sexual and reproductive health and rights (SRHR) and FP? Fortunately, we have a history to draw on. From the Alma Ata Declaration of 1978 to the 1994 International Conference on Population and Development (ICPD), to the 2000 ICESCR Article 12 General Comment No. 14 and, most recently, the Nairobi Summit on ICPD + 25, the global health community has come together in the past to set out a vision that centres people and their rights to participate in the health decisions that affect them and to determine their own reproductive futures. Over the years there have been remarkable gains; we have moved from a primary focus on population issues and fertility to one grounded in the rights of individuals to control their bodies and attain reproductive health and well-being, but we are yet to fully achieve this vision. Progress has been impeded in part by a failure to fully articulate, commit to and operationalise these values.

FP programming is still often framed around a notion of supply and demand that juxtaposes ensuring adequate availability of contraceptive services and supplies with generating interest and demand for those services. This frame, with its prescriptive expectation that people should use contraception, fails to take into account people's self-expressed needs, values, and preferences, runs counter to the goal of reproductive rights, and disregards the personal, social, and structural forces that influence behaviour. Further, FP programmes often myopically focus on contraceptive information, products, and services without considering the broader context of poverty, limited economic and educational opportunities, gender inequality, racism, and harmful social norms, all of which constrain choice, inhibit people's ability to realise, or even imagine, a range of possible futures, and block the achievement of one's reproductive goals. And, despite some important efforts and incremental progress, the global FP community continues to measure success of FP programmes by increases in the modern contraceptive prevalence rate, or reductions in contraceptive discontinuation, measures that at best fail to capture reproductive power and agency and, at worst, contribute to impeding them. Even more concerning is the resurfacing of narratives, and resulting programmes and policies, that instrumentalise FP as a solution to climate change, resource scarcity, poverty, and migration; narratives that not only undermine people's reproductive power, but also ultimately fail to address the underlying causes that drive all of these injustices. Further, when these issues are catastrophised, as climate change increasingly is, solutions that abrogate rights are more likely to be seen as justifiable.

To protect and accelerate progress, now is the time for a value-driven evolution in global FP work. The reproductive justice movement points the way. While founded in human rights principles, reproductive justice goes beyond what the FP community calls “voluntary rights-based family planning”^5^ which, while a significant advance, still largely positions FP as an offset (separated from the other domains of reproductive life course decisions) and has thus far failed to engender truly transformative approaches and metrics. Reproductive justice is defined as “the human right to maintain personal bodily autonomy, have children, not have children, and parent the children we have in safe and sustainable communities”, and is focused on addressing structural inequalities and dismantling intersecting spheres of oppression that constrain individuals’ reproductive agency.^3^ This movement has transformed the way reproductive health services and contraceptive counselling are conceptualised in the US,^6,7^ if not yet fully transformed the delivery of those services. The emergence of these concepts in the broader global health discourse is exciting: the time is right for the global FP community to rethink how we do what we do, what we truly value, and how we might more fully imagine the future. We have an opportunity to collectively articulate a new value-based framing for our work with clear guiding principles, language, and metrics, to drive transformational progress. In hopes of advancing this conversation about the values, goals, principles, and focus of our global FP work, we offer the Family Planning Ecosystem Framework,^8^ described below, as one step in that direction.

## The family planning ecosystem framework

Reproductive power – the ability to make and act on one's reproductive choices – is put forward as the value-based goal of FP work in this framework. When a person recognises their social position and the conditions that shape their sexual and reproductive choices, they can begin to think differently, challenge assumptions and norms, and imagine new possibilities (critical consciousness), including the possibility of working with others to change social and material conditions that constrain their choices. From this position, they can better navigate the relationships and structures that may impede their ability to act on their reproductive desires and take purposeful action to achieve their reproductive goals. We want to emphasise that reproductive power is not about achieving the demographic dividend or adapting to climate change. Reproductive power is about a person's ability to make and act on their reproductive choices not just once, but repeatedly as they make their way through life – as they move from adolescence to adulthood, face changes and challenges in their relationships; in their physical, social, or economic conditions; and in their experience with the health delivery system, whether public or private or digital.

The framework embraces the idea of a dynamic, interconnected ecosystem of individual, relational, and structural elements that interact to facilitate or impede a person's reproductive power. Driving toward reproductive power requires building and fostering individuals’ capabilities, assets, and expectations, as well as conditions conducive to this in both the community and the health delivery system. On the community side of the equation, building and fostering these elements – through, for example, raising awareness, facilitating dialogue and reflection, building self-efficacy, and removing social, structural, and material constraints – will lead to individuals and couples who are better able to exercise agency and navigate their own FP path. On the health delivery side, building and fostering these elements – through, for example, fully equipping and empowering health workers, aligning expectations and incentives, and increasing support and participatory governance – enhance the health system's ability to adapt and deliver quality FP equitably and sustainably. Ensuring people are better able to navigate their FP journey, and ensuring the health system is better able to respond, will increase the likelihood that the interaction between the individual and the health system will be positive, and support individuals’ reproductive power. Furthermore, this kind of productive, generative interaction may help to create a self-improving health system, one in which both the community and health delivery system see their engagement as producing valued change, thus generating more positive expectations of, and trust in, the system and motivation to seek continued improvements.

The framework is underpinned by three key principles: people, power, and connection:

### People

This principle suggests we focus programme design, innovation, and improvement around people and relationships, always keeping women, girls, and marginalised people at the centre of our efforts. Health systems are more than commodities and infrastructure, “health systems are also human systems”.^9^ Many different people make up health systems – policy makers, service providers, service users – and have various roles, stakes, and power within them. As public health crises such as the 2013–2016 Ebola outbreak in West Africa illustrate, over-reliance on technical solutions without the engagement and support of actors in the system, including affected populations and communities, dooms them to failure. It was not until women and communities were engaged in the Ebola response, e.g. in tracking and addressing rumours, that the outbreak was brought under control.^10^ Focusing on new technologies and the hardware of the health system underestimates the dynamic human dimensions that drive behaviour, trust, motivation, quality, positive user experience, and, ultimately, positive programme outcomes.

### Power

A person's ability to make and act on their FP decisions is complex and encompasses the continually negotiated relationships and power dynamics within the household, the community, and between users and the health delivery system. It is impossible to inventory all the unique conditions, narratives, beliefs, practices, resource constraints, and dynamics that a person might face at any given time in any given context, nor do we have the resources to tailor interventions for every context and condition – particularly since those contexts and conditions change constantly. As programme designers and researchers, rather than trying to identify and “solve for” every possible barrier or challenge a person might face, this principle suggests we place greater emphasis on “solving with”. In other words, work should focus on creating the conditions that will support an individual's capacity to identify, analyse, and overcome the interpersonal, social, structural obstacles that they face across their reproductive life journey, so they can exercise their power to navigate their own path. Further, the actors within the health delivery system need to be equipped to respond and adapt to users’ needs and preferences and to ever-changing conditions. We need to reshape power relations by ensuring people at all levels of the health system – patients, family, community members, and providers – have real voice and agency in the creation of health and well-being. This approach has the potential to create a virtuous cycle, as clients are more able to negotiate services, demand quality, and claim their rights to respectful, equitable care, and health providers and the delivery system are better able to adapt and respond effectively to those claims.

### Connection

Related to shifting power dynamics, the third principle underpinning work to support an individual's ability to make and act on their FP decisions should be a focus on connection, participation, and engagement. The global FP community has largely approached the community system and health system as two siloed systems. However, there is a dynamic relationship between these two systems and, as such, efforts are needed to strengthen the connection between the two by fostering social participation and engagement. There is growing realisation that social participation in health care is not only a human right, but also holds value in improving health care and keeping systems accountable. Community members are experts in their context and experience and can deploy this knowledge to help solve health care problems. Mechanisms that bring community members and health providers together to mutually identify service provision and utilisation problems, and then jointly generate, negotiate, and monitor solutions, have been successful in improving access, utilisation, quality, and governance of services.^11,12^ To achieve the goal of reproductive power, we need to pay more attention to these connections and the mechanisms that enable and support participation, engagement, user and community voice, system responsiveness, adaptive capacity, and quality.

## Conclusion

The Ecosystem Framework is not meant to be a rigid prescription, nor are we advocating for the wholesale adoption of this framework by the global FP community. Rather, we hope that this framework spurs a deeper conversation about a value-driven evolution of global FP work. Not having a strong value base makes it hard to challenge repressive narratives and to articulate why a focus on the benefits of FP to outcomes other than increasing people's reproductive power and agency, is dangerous. Focusing on how FP can support adaptation to climate change or increase “human capital” and economic development, not only unfairly places the burden of solving these problems on poor people but diverts attention from the global inequities in resource consumption, health care access, and political power that drive these intersecting injustices. FP donors and programme implementers often resist calls to address these broader issues, saying these areas are not our “lane”; in doing so, however, we miss perhaps the biggest, most transformative opportunities to accelerate progress. We hope the Family Planning Ecosystem Framework, along with other recent frameworks and calls to action, catalyses conversation and action, and moves us to a new, transformed, ecosystem that is focused on creating the conditions that will truly enable reproductive power, health, and well-being for all.

While turbulent, the moment we are in has made it clear that reimagining our approach to global FP work is not only desirable, but necessary. The resurfacing of repressive narratives and advocacy agendas that cast FP as a solution to resource stress, migration, social instability, or rising carbon emissions, are red flags: we need to take heed. A new framework, born of a robust and sincere grappling with the issues that underpin and drive global injustices, could provide the language and the tools to ensure that we stay focused on root causes and mechanisms, understand the context and the dynamics, and prioritise reproductive power and agency. Inspired by the reproductive justice movement and a growing recognition of the intersecting injustices we must address as a global FP community, we hope that the ecosystem framework described here can be a valuable input into this process. Ultimately, however, the global SRHR/FP community needs to come together with leaders spanning reproductive, climate, gender, economic, and racial justice, to co-create and articulate a new value-based frame with clear principles, language, and metrics. We are getting closer, but we are not yet there: it is time to align our actions with our values.
